# Upregulated IL-6 Indicates a Poor COVID-19 Prognosis: A Call for Tocilizumab and Convalescent Plasma Treatment

**DOI:** 10.3389/fimmu.2021.598799

**Published:** 2021-03-04

**Authors:** Jian Wu, Jiawei Shen, Ying Han, Qinghua Qiao, Wei Dai, Bangshun He, Rongrong Pang, Jun Zhao, Tao Luo, Yanju Guo, Yang Yang, Qiuyue Wu, Weijun Jiang, Jing Zhang, Mingchao Zhang, Na Li, Weiwei Li, Xinyi Xia

**Affiliations:** ^1^COVID-19 Research Center, The First School of Clinical Medicine, Institute of Laboratory Medicine, Jinling Hospital, Nanjing University School of Medicine, Southern Medical University, Nanjing, China; ^2^Medical and Technical Support Department, Pingdingshan Medical District, The 989th Hospital Pingingshan, Pingdingshan, China; ^3^Joint Expert Group for COVID-19, Department of Laboratory Medicine & Blood Transfusion, Wuhan Huoshenshan Hospital, Wuhan, China; ^4^General Clinical Research Center, Nanjing First Hospital, Nanjing Medical University, Nanjing, China; ^5^Department of Laboratory Medicine, Nanjing Red Cross Blood Center, Nanjing, China; ^6^Institute of Blood Transfusion, Jinling Hospital, Nanjing University School of Medicine, Nanjing, China; ^7^Department of Blood Transfusion, Jingling Hospital, Nanjing University School of Medicine, Nanjing, China

**Keywords:** SARS-CoV-2, COVID-19, IL6, tocilizumab, CPT, discharge

## Abstract

A comprehensive understanding of the dynamic changes in interleukin-6 (IL-6) levels is essential for monitoring and treating patients infected with severe acute respiratory syndrome coronavirus 2 (SARS-Cov-2). By analyzing the correlations between IL-6 levels and health conditions, underlying diseases, several key laboratory detection indices, and the prognosis of 1,473 patients with the coronavirus disease 2019 (COVID-19), the role of IL-6 during SARS-CoV-2 infection was demonstrated. Our results indicated that IL-6 levels were closely related to age, sex, body temperature, oxygen saturation (SpO_2_) of blood, and underlying diseases. As a stable indicator, the changes in IL-6 levels could indicate the inflammatory conditions during a viral infection. Two specific treatments, namely, tocilizumab and convalescent plasma therapy (CPT), decreased the level of IL-6 and relieved inflammation. CPT has an important role in the therapy for patients with critical COVID-19. We also found that patients with IL-6 levels, which were 30-fold higher than the normal level, had a poor prognosis compared to patients with lower levels of IL-6.

## Introduction

As of late August 2020, the coronavirus disease 2019 (COVID-19) has been confirmed in over 20 million people worldwide, carrying a higher mortality rate than influenza, which has a mortality rate lower than 1% (1). Compared to other coronaviruses, such as severe acute respiratory syndrome coronavirus (SARS-CoV), which caused an outbreak of SARS in 2003, the transmission capacity of SARS-CoV-2 is much stronger (2). It is an extremely serious task to prevent and control COVID-19 due to the rapid increase in confirmed cases. An effective treatment is also needed urgently. As preventative vaccines and effective antivirals remain unavailable, host-directed therapeutics employing existing immunomodulatory agents should be explored (3).

The spectrum of clinical syndromes for patients with COVID-19 range from flu-like, mild illness to severe pneumonia (4). A series of symptoms are found with SARS-CoV-2 infections, including fever, cough, myalgia or fatigue, dyspnea, acute cardiac injury, acute respiratory distress syndrome (ARDS), and secondary infection; some patients with severe COVID-19 have been admitted to the intensive care unit (ICU) (5, 6). Accumulating evidence has suggested that the percentage of patients with severe COVID-19 might have cytokine storm syndrome (CSS) (1). Inflammation, indicated by elevated plasma levels of several proinflammation cytokines, such as interleukin-6 (IL-6), interleukin-1 beta (IL-1β), and tumor necrosis factor-alpha (TNF-α), is also thought to indicate severe COVID-19 (6). IL-6, a key mediator of the immune pathway, plays an important role in the cytokine network and acute inflammation (7). Hyperactivation of IL-6 can cause respiratory failure, shock, and multiorgan dysfunction (8).

Here, we present an analysis of IL-6 levels related to several aspects of SARS-CoV-2 infection in 1,472 patients with COVID-19, hospitalized at the Wuhan Huoshenshan Hospital. The changes in their IL-6 levels were connected to baseline information, underlying diseases, results of nuclear acid detection, routine laboratory detection, multiple cytokines, antibodies, and lymphocyte subsets. The effects of two important therapy methods, namely, the tocilizumab and convalescent plasma therapy (CPT) on IL-6 levels were also analyzed, which demonstrated that both methods were beneficial to patients with COVID-19, as indicated by a decrease in IL-6 levels. The prognoses for patients with high levels of IL-6, who received treatment with tocilizumab and CPT, were analyzed. These results provide a comprehensive understanding of the dynamic changes and key roles of IL-6 throughout a SARS-CoV-2 infection.

## Methods

### Patient Involvement and Data Collection

A total of 1,472 hospitalized patients (admitted from February 4, 2020 to March 30, 2020 in the Huoshenshan Hospital), who were clinically diagnosed and laboratory confirmed to have a COVID-19 infection, were preliminarily involved in this study. The clinical outcomes were monitored till April 14, 2020. Patients were classified into moderate, severe, and critical groups based on the novel coronavirus pneumonia diagnosis and treatment guideline from China (7th edition). Specifically, the patients with COVID-19 who met any of the following three criteria were defined as severe patients: (1) respiratory distress ≥30 breaths per minute; (2) oxygen saturation (SpO_2_) at rest less than or equal to 93%; (3) arterial partial pressure of oxygen (PaO_2_)/fraction of inspired oxygen (FiO_2_) ≤ 300 mm Hg. Patients whose chest imaging showed obvious lesion progression within 24–48 h larger than 50% were also defined as severe cases. Patients who reach any of following criteria were defined as critical: (1) shock, (2) respiratory failure and requiring mechanical ventilation, (3) organ failure that requires ICU monitoring and treatment.

Clinical characteristics, including medical history, comorbidities, surgical history, disease history, and symptoms, were collected. Treatment and outcome data for each patient were also obtained from the electronic medical record system of the Huoshenshan Hospital with a standardized data collection form. This study was a descriptive-correlative study and approved by the Medical Ethical Committee of Wuhan Huoshenshan Hospital of China, and a written informed consent was obtained from each patient.

### Laboratory Testing

The confirmation of a SARS-CoV-2 infection in the laboratory has been described in previous reports (9, 10). The test samples were sent to a designated agency that performed the testing. Briefly, throat-swab specimens were obtained and total nucleic acids were extracted. The ORF1ab and nucleocapsid genes were detected. A real time (RT)-PCR was performed to confirm the presence of the virus. The number of amplification of cycle threshold values (Ct) was used to indicate the viral load. The Ct values of ORF1ab and N genes lower than 40 were defined as positive.

Total SARS-CoV-2 immunoglobulin M (IgM) and IgG in the serum were measured using chemiluminescence with a commercial detection kit (Shenzhen YHLO Biotech Co., Ltd, China, 20200206). The levels, RBD-specific, S-specific, and N-specific IgM and IgG were detected using chemiluminescence, with a commercial kit, according to the instructions of the manufacturer (Nanjing RealMind Biotech Co., Ltd, China RBD-IgM: R90320022001; RBD-IgG: 90420022001S-IgM: R90120022001; S-IgG: R90220022001; N-IgM: R90520022001; N-IgG: R90620022001). Shortly, blood was centrifugated at room temperature, and the supernatants were collected and incubated with SARS-CoV-2 antigen-coated magnetic beads. The antigen–antibody complex was then captured by the beads and followed by a gentle separation using a magnetic rack. The complex was then incubated with an acridinium-ester-labeled mouse anti-human IgG or IgM antibody and then reacted with hydrogen peroxide in an excitation buffer. Finally, relative luminescence intensity was measured using an ACL2800 chemiluminescence system (Nanjing RealMind Biotech Co., Ltd, China).

For each patient, routine blood examinations included a complete blood count, coagulation profile, and serum biochemical tests (including renal and liver function, creatine kinase, lactate dehydrogenase, and electrolytes). Briefly, the plasma samples of patients were analyzed with a chemiluminescent immunoassay (CLIA) based on the SAL 9000 Modular System with a CLIA kit, which was supplied by Shenzhen Mindray Bio-Medical Electronics Co., Ltd. (Shenzhen, China) according to the instructions of the manufacturer.

The cytokines and chemokines (IL-5, IFN-α, IL-2, IL-6, IL-1β, IL-10, IFN-γ, IL-8, IL-17, IL-4, IL-12p70, and TNF-α) of plasma samples from patients with COVID-19 were measured using a microsphere flow immunofluorescence, according to the instructions of the manufacturer of the commercial kit supplied by Qingdao Raisecare Biotechnology Co. Ltd, China. Briefly, blood samples were centrifuged at room temperature, and the plasma was collected and incubated with capture microspheres, which were coated with matched antibodies. The antigen–antibody complex, captured by the microspheres, was then incubated with biotin-labeled detection antibodies; later, phycoglobin-labeled streptavidin microspheres were added. Then a phosphoric acid buffer with bovine serum albumin (BSA) was added to the reactant. Phycoerythrin (PE) fluorescence intensity was measured using Beckman Dxflex Flow cytometry (Beckman Coulter, Inc., USA). The PE fluorescence intensity was converted to the concentration of cytokines and chemokines based on the standard curve.

The lymphocyte subgroups were measured using Flow cytometry (CytoFLEX flow cytometry system, Beckman Coulter, Inc.), with a commercial kit (Beckman coulter, Inc.), according to the protocol of the manufacturer.

### Study Outcomes

The improvement of clinical symptoms of each patient was evaluated by a six-category scale score (SCSS), which was described in a previously published literature (blood). The SCSS contained the following levels: (1) patients discharged,; (2) patients were still hospitalized, but not requiring oxygen therapy; (3) patients were hospitalized and requiring low-flow oxygen therapy; (4) patients were hospitalized and requiring high-flow oxygen therapy, non-invasive mechanical ventilation or both methods; (5) patients were hospitalized and requiring extracorporeal membrane oxygenation (ECMO) invasive mechanical ventilation or both; (6) death. For each patient, the SCSS was evaluated and updated each day.

### Statistical Analysis

Statistical analyses were performed using the R software (version 3.5.2) and GraphPad Prism version 8.00 software (GraphPad Software Inc.). Graphs were generated and plotted using the software, GraphPad Prism version 8.00 and the R software (version 3.5.2). The Mann–Whitney *U*-test was used for two independent samples and the Kruskal–Wallis test was used to assess multiple group differences; values of *p* < 0.05 were considered statistically significant.

## Results

### The Relationship Between IL-6 Levels and Health Conditions of Patients

**Figure 1:**
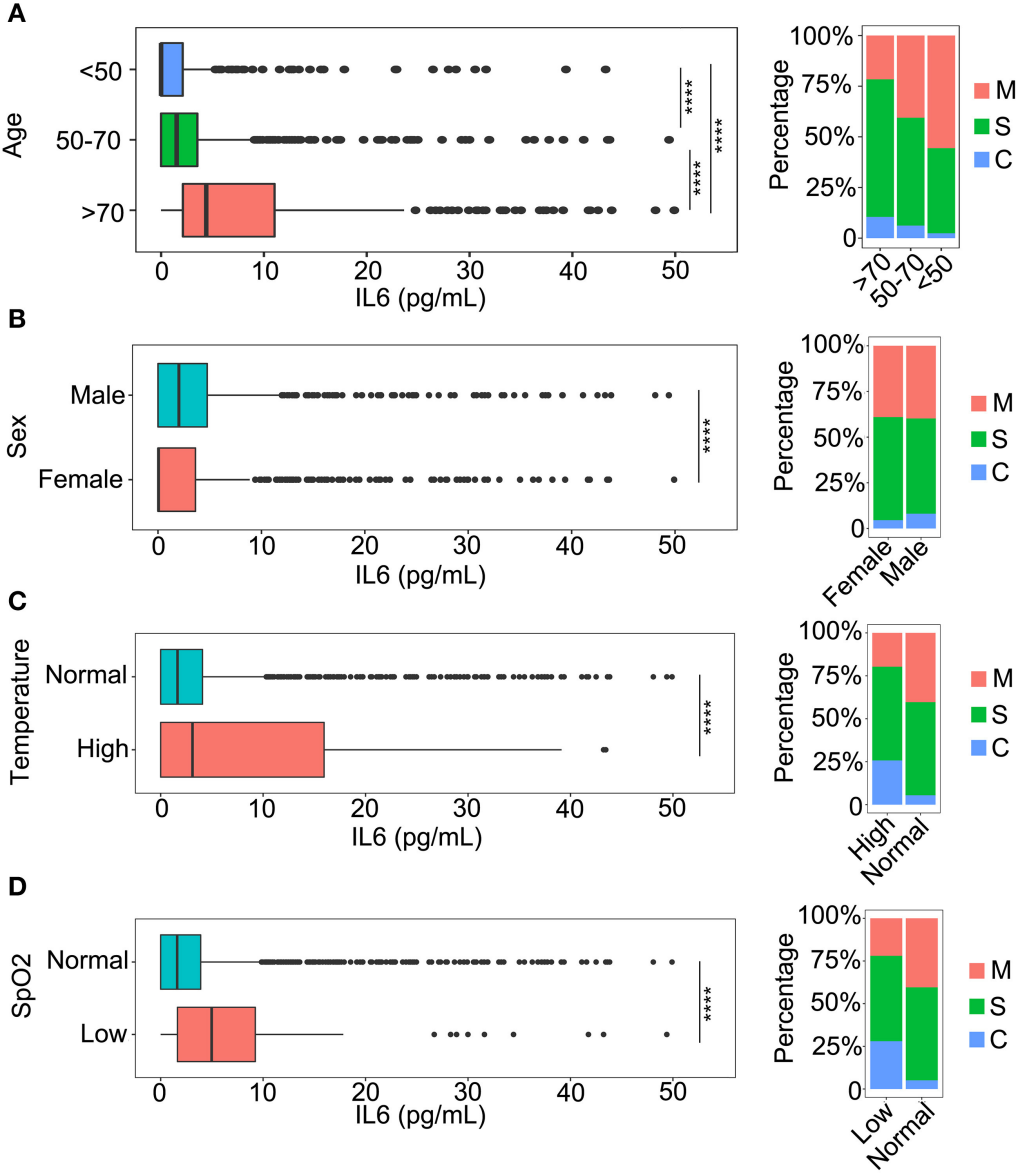
The relationship between interleukin-6 (IL-6) levels and baseline information. **(A)** A comparison of IL-6 levels among three age categories and the classification of patients in each category: <50 years, patients younger than 50 years old; 50–70 years, the age of patients older than 50 and younger than 70; and >70 years, patients older than 70 years. M, mild patients; S, severe patients; C, critical patients. ****p < 0.001. **(B)** The comparison of IL-6 levels between males and females. M, mild patients; S, severe patients; C, critical patients. ****p < 0.001. **(C)** The comparison of IL-6 levels in patients with normal temperature and patients with fever. Normal, patients with temperature lower than 37.3°C; High, patients with temperature higher than 37.3°C. M, mild patients; S, severe patients; C, critical patients. ****p < 0.001 **(D)** The comparison of IL-6 levels between normal and oxygen deficit patients. Normal, patients with oxygen saturation (SpO_2_) higher than 93%; Low, patients with SpO_2_ lower than 93%. M, mild patients; S, severe patients; C, critical patients. ****p < 0.001.

To investigate the relationship between IL-6 and the demographic characteristics of patients, a comparison of several aspects of the patients was performed. Since age is an important factor related to infection with COVID-19, the patients were divided into three groups (<50 years; 50–70 years; >70 years) according to the age distribution of all patients (Figure 1A, Supplementary Figure 1). The comparison results indicated that the level of IL-6 was increased with an increase in age (Figure 1A), and that the group containing patients who were older than 70 years had the highest level of IL-6 (Figure 1A). In addition, the severity of the COVID-19 infection was closely related to an increase in age (Figure 1A). The levels of IL-6 were also compared between different genders of patients. The levels of IL-6 in male patients were higher than that of females (Figure 1B), which might be associated with the fact that male patients are more critically ill (Figure 1B). The temperatures of the patients were considered as an important feature of COVID-19 infection. As a result, the relationship between temperatures and IL-6 levels were also compared. The patients who had a temperature above 37.3°C had higher IL-6 levels compared to the patients with normal temperature (Figure 1C). Moreover, the severity of the disease was also closely related to the body temperature (Figure 1C). In the oxygen saturation (SpO_2_) of blood, the level of SpO_2_ was negatively correlated with the level of IL-6 (Figure 1D). The severity of the disease was associated with a decrease in SpO_2_, which might be related to an increase in the level of IL-6 (Figure 1D).

### The Relationship Between IL-6 and the Underlying Diseases of Patients With COVID-19

**Figure 2:**
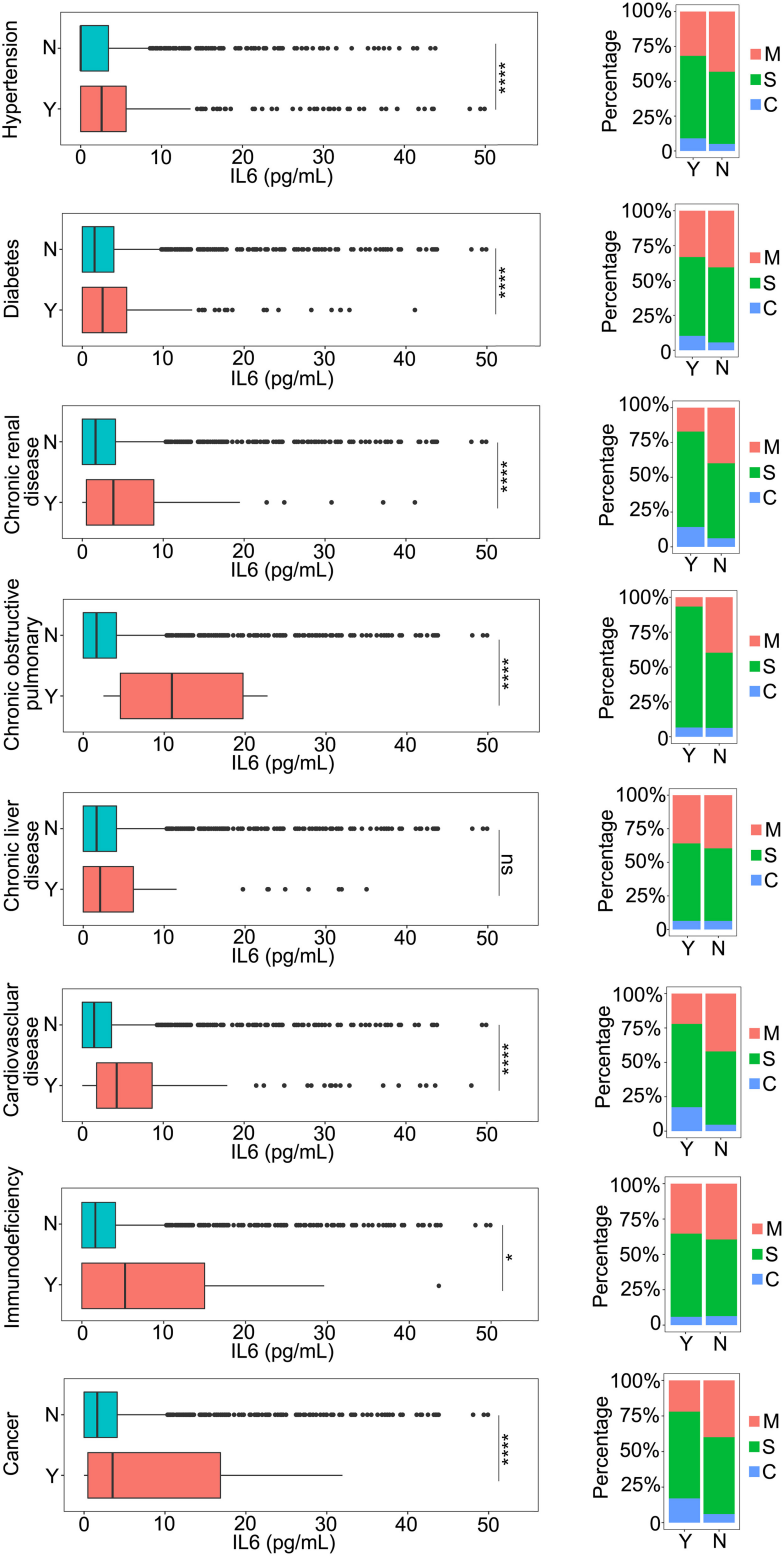
The relationship between IL-6 and the underlying diseases. The IL-6 levels of patients with or without underlying diseases were compared. The percentage of coronavirus disease 2019 (COVID-19) classifications in each group was compared. N, patients without underlying disease; Y, patients with underlying disease M, mild patients; S, severe patients; C, critical patients. *p < 0.05; ****p < 0.001; ns, not significant.

To detect the effect of the underlying diseases during therapy for patients with COVID-19, the relationship between the underlying diseases and the levels of IL-6 was determined. Hypertension and diabetes, the most common underlying diseases, were closely related to disease progression in the patients (11–14), which could be associated with the level of IL-6 (Figure 2). During the progression of the disease, all patients with hypertension or diabetes were found to have higher levels of IL-6 compared to patients without these comorbidities (Figure 2). In addition, we observed that the severity of the disease was also related to these two kinds of comorbidities (Figure 2), and patients with hypertension or diabetes had a higher risk of disease progression (Figure 2). Moreover, the results revealed that several kinds of chronic disease, such as chronic renal disease, chronic obstructive pulmonary disease, and chronic liver disease, could also increase the level of IL-6 and the severity of the COVID-19 infection (Figure 2), and that patients with cardiovascular disease had higher IL-6 levels and were susceptible to disease progression (Figure 2). Immunodeficiency, another important aspect of an underlying disease, was also analyzed with the level of IL-6. The inflammation caused by COVID-19 infection could also be increased by immunodeficiency (Figure 2). The role of cancer during the infection process was also analyzed. As shown in Figure 2, patients with cancer had a more serious inflammation condition as indicated by a higher level of IL-6 (Figure 2). Based on these comparison results, several kinds of underlying diseases could be associated with an increase in the severity of inflammation caused by the COVID-19 infection.

### The Relationship Between IL-6 Levels and Clinical Laboratory Test Items

**Figure 3:**
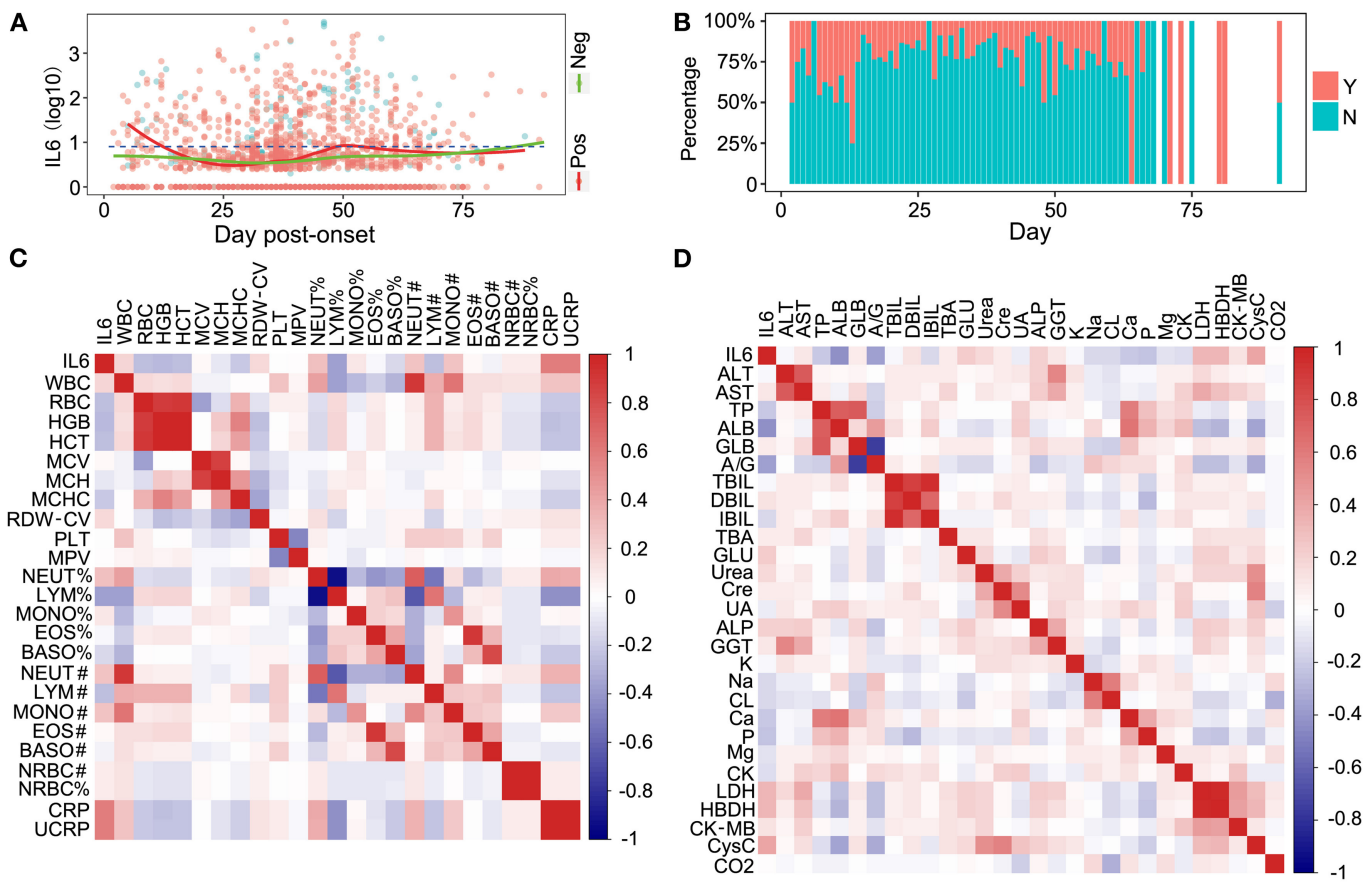
The relationship between IL-6, nuclear acid detection, and routine laboratory detection. **(A)** The relationship between IL-6 and SARS-Cov-2 nuclear acid detection. Pos, nuclear acid detection positive; Neg, nuclear acid detection negative. **(B)** The comparison of inflammatory conditions and severe acute respiratory syndrome coronavirus 2 (SARS-Cov-2) acid clearance times. Y, patients with inflammation; N, patients without inflammation. **(C)** The correlation between IL-6 and blood routine test items. **(D)** The correlation between IL-6 and biochemical routine test items.

The clinical laboratory test items are the most important indicators in the progress of therapy in patients with COVID-19. They can be used for monitoring the condition of patients in a timely and accurate manner. To define the relationship between the IL-6 levels and potential clinical laboratory test items, a correlation analysis was performed. Nucleic acid detection is the most important diagnostic factor and it confirms the features of COVID-19 infection. The relationship between the detection results and the level of IL-6 was analyzed. Patients with negative nucleic acid detection results had lower IL-6 levels compared to the patients with positive detection results, indicating that they had a lower level of inflammation (Figure 3A). The IL-6 levels of patients with negative nucleic acid results were slightly increased when the days post onset (dpo) were longer than 75 days (Figure 3A), and this might be caused due to a long term infection and several methods of medical treatment. The relationship between IL-6 levels and the day that reflected the negative nucleic acid result was also investigated. Patients with SARS-CoV-2, whose respiratory tracts were cleared in <12 days, might have had a higher level of inflammation as indicated by a high level of IL-6 (Figure 3B). When the nucleic acid result became negative in <2 months, the inflammation in patients was controlled, and the percentage of patients without inflammation was higher than the percentage of patients who still had inflammation (Figure 3B). Patients with longer times to a negative status had higher levels of IL-6; however, this might have been due to the long hospitalization time and long therapy process (Figure 3B). Routine blood tests were commonly used for detection during the therapy procedure. The relationship between IL-6 and all routine blood test items was determined and C-reaction protein (CRP) and ultra C-reaction protein (UCRP) were all closely related to the level of IL-6 (Figure 3C). The above three indices were all used to indicate the level of inflammation. In addition, indices from routine blood tests, such as the monocyte count (MONO#), the neutrophil count (NEUT#), and total white blood cells (WBCs), all showed a positive correlation with IL-6 levels (Figure 3C). The correlation between IL-6 and routine biochemical test items were also detected. Several indices related to different tissues showed a positive correlation with IL-6 levels, such as alkaline phosphatase (ALP), which is related to the liver, and cystatin C (CysC), which is related to the kidney (Figure 3D). There were also some indices that showed a negative correlation with IL-6 levels, such as albumin (ALB), which is not closely related to inflammation (Figure 3D). Moreover, an infection of COVID-19 could also cause multiple organ injuries, including the heart, kidney, and liver, which could be demonstrated through clinical laboratory test items (Supplementary Figure 3). Coagulation indices were also closely related to COVID-19 infections (Supplementary Figure 4). Coagulation indices could be used to reflect the order of severity of the disease. Based on these results, we deem that the level of IL-6 was closely related to several clinical laboratory test items.

### The Relationship Between IL-6 and Multiple Cytokines

**Figure 4:**
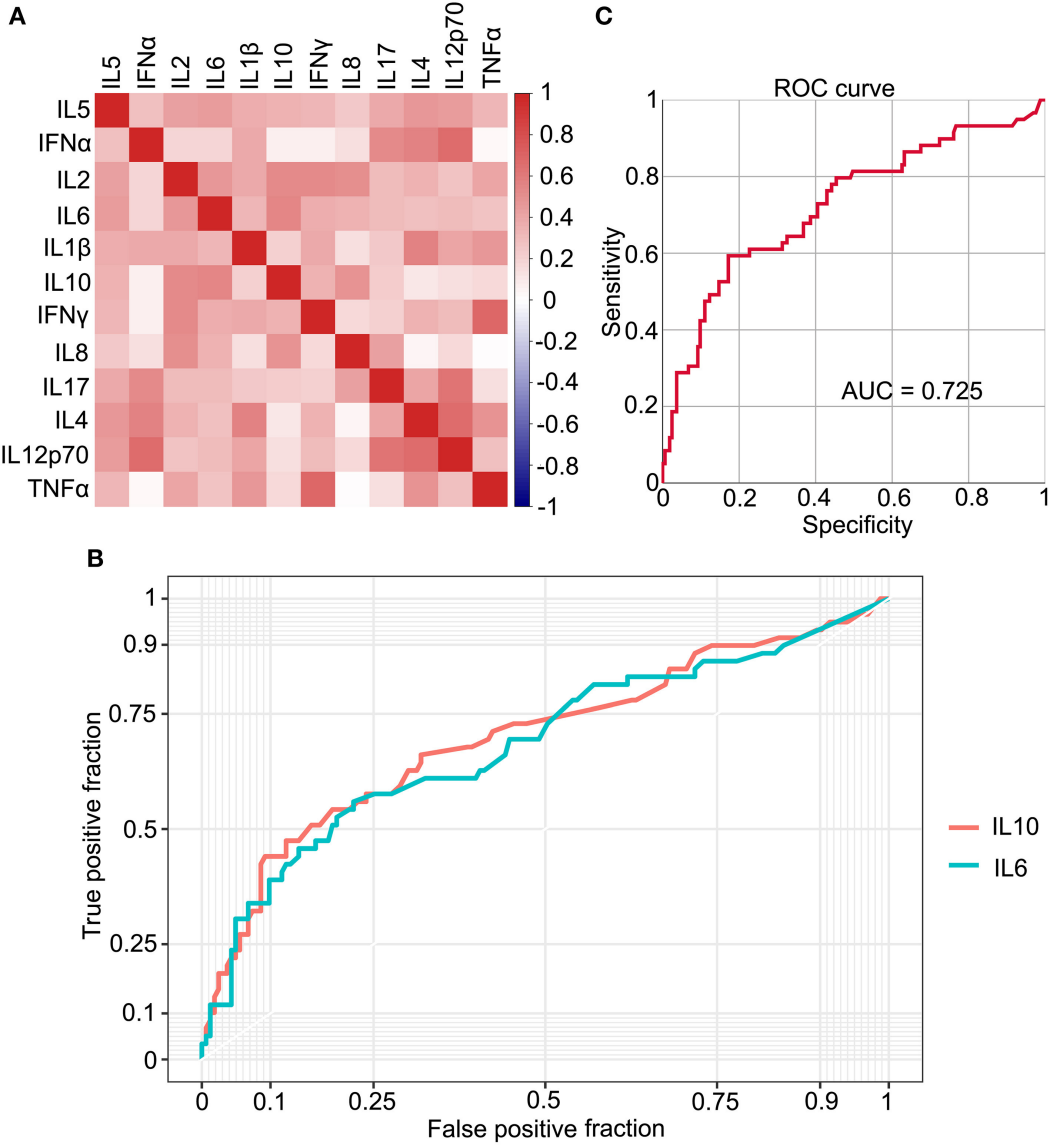
The relationship between IL-6 and multiple cytokines. **(A)** The correlation between 12 types of cytokines. **(B)** The receiver operating characteristic (ROC) curve plots IL-6 and IL-10, individually. **(C)** The ROC curve plots based on IL-6 and IL-10.

Cytokines serve an important role in the inflammation process during the SARS-Cov-2 infection. To investigate the relationship between IL-6 and other cytokines, 12 kinds of cytokines were detected in patients with COVID-19. IL-6, a key factor of inflammation, was positively correlated with several cytokines, although the correlation coefficients of some cytokines were low (Figure 4A). IL-10 was positively correlated with IL-6, which had the highest correlation coefficient, and a receiver operating characteristic (ROC) curve was plotted (Figure 4B). The area under the curve (AUC) for both the cytokines was ~0.7 (IL-6, 0.686; IL-10, 0.701), which is a higher value than others (Figure 4B and Supplementary Figure 2). To improve the sensitivity and specificity of IL-6 and IL-10 for a progression prediction, an ROC curve was plotted using these two cytokines, and the feature of the ROC curve was improved slightly (AUC = 0.725) (Figure 4B). Based on these results, multiple cytokines could be detected during the COVID-19 infection, and IL-6 could be used as an excellent biomarker to reflect the level of inflammation directly and easily.

### The Different Dynamic Changes in IL-6 and Antibodies

**Figure 5:**
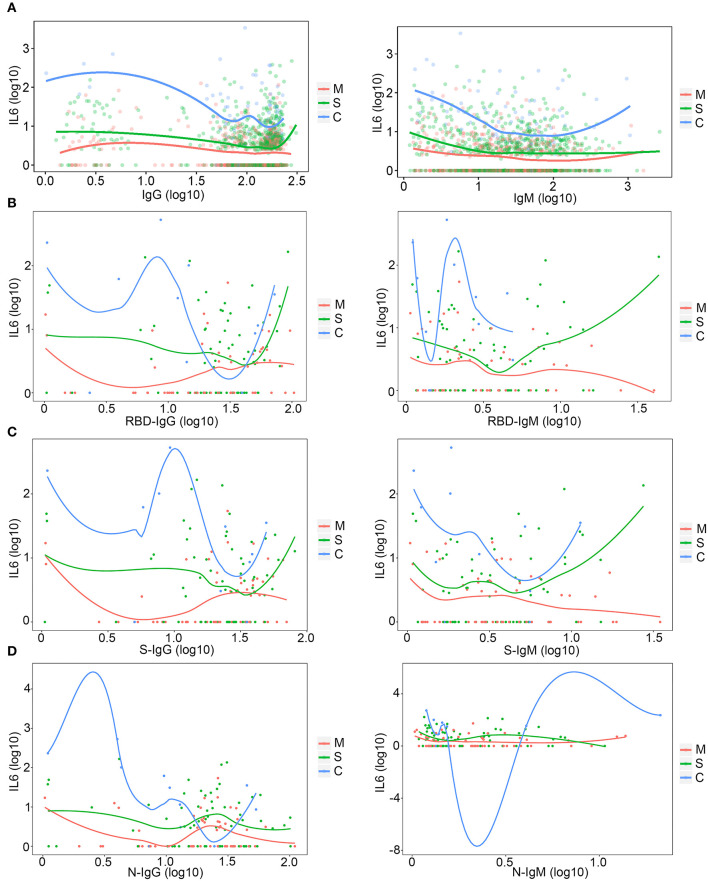
The relationship between IL-6 levels and antibodies. **(A)** The relationship between IL-6 and total immunoglobulin G (IgG) and IgM. M, mild patients; S, severe patients; C, critical patients. **(B)** The relationship among IL-6, RBD-IgG, and RBD-IgM. M, mild patients; S, severe patients; C, critical patients. **(C)** The relationship among IL-6, S-IgG, and S-IgM. M, mild patients; S, severe patients; C, critical patients. **(D)** The relationship among IL-6, N-IgG, and N-IgM. M, mild patients; S, severe patients; C, critical patients.

The total amount of IgM and IgG antibodies are key features during a SARS-CoV-2 infection and during the therapy for patients with COVID-19. To explore whether total antibody levels could be changed along with changes in inflammation, the level of antibodies was compared under different IL-6 levels. With an increase in the IL-6 level, total IgG levels in patients with mild, severe, and critical COVID-19 infection vary (Figure 5A). Specifically, the level of IgG increased with the order of severity; patients with a critical SARS-CoV-2 infection had the highest level of IgG (Figure 5A), and the levels of IgG in patients with both critical and mild COVID-19 were decreased with an increase in the IL-6 level, which might be caused by the exhaustion of the neutralizing antibody. The IgG level in severe patients increased when the patients suffered with serious inflammation (Figure 5A). The level of IgM of all categories of patients showed the same trend (Figure 5B). The level of total IgM in all three categories of patients increased with high IL-6 levels (Figure 5B), and the level of IgM was also positively correlated with the severity of the disease (Figure 5B). The receptor-binding domain (RBD) specific antibodies were also compared to the level of IL-6. Both RBD-IgG and RBD-IgM showed distinctive trends with increase in IL-6 levels (Figures 5C,D), which might indicate the different roles of these antibodies in different inflammatory conditions.

### Lymphocytes Were Decreased With Increase in IL-6

**Figure 6:**
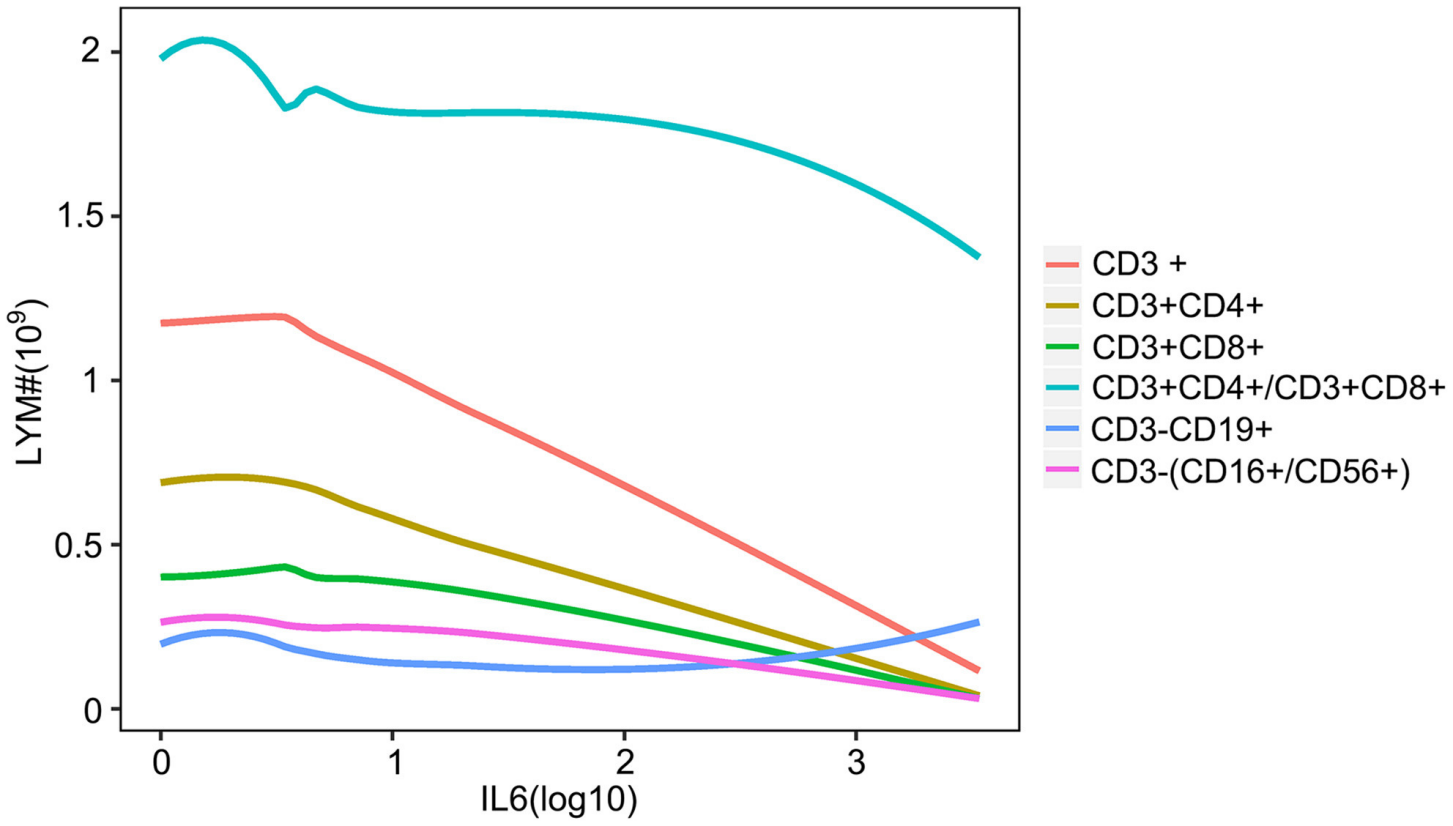
The relationship between IL-6 and lymphocyte subsets. The line plot indicates the dynamic changes in lymphocyte subsets with increase in the level of IL-6. Different colored lines show the different lymphocyte subsets. CD3+, CD3 positive lymphocyte; CD3+CD4+, CD3 and CD4 positive lymphocyte; CD3+CD8+, CD3 and CD8 positive lymphocyte; CD3+CD4+/CD3+CD8+, CD3, CD4, and CD8 positive lymphocyte; CD3-CD19+, CD3 negative and CD19 positive lymphocyte; CD3-(CD16+/CD56+), CD3 negative, CD16, and CD56 positive lymphocyte.

The disease process and the severity of infection in patients with COVID-19 were associated with the dynamic changes in the number of lymphocytes. To investigate the relationship between lymphocytes and IL-6 levels, the dynamic trend of lymphocytes along with IL-6 was analyzed. As shown in Figure 6, T- and NK-cell subsets were decreased with an increase in IL-6, except for B lymphocytes (Figure 6). The number of B lymphocytes also decreased with slight inflammation, while patients with higher inflammatory conditions had larger number of B lymphocytess (Figure 6).

### IL-6 Decreases With Tocilizumab Treatment

**Figure 7:**
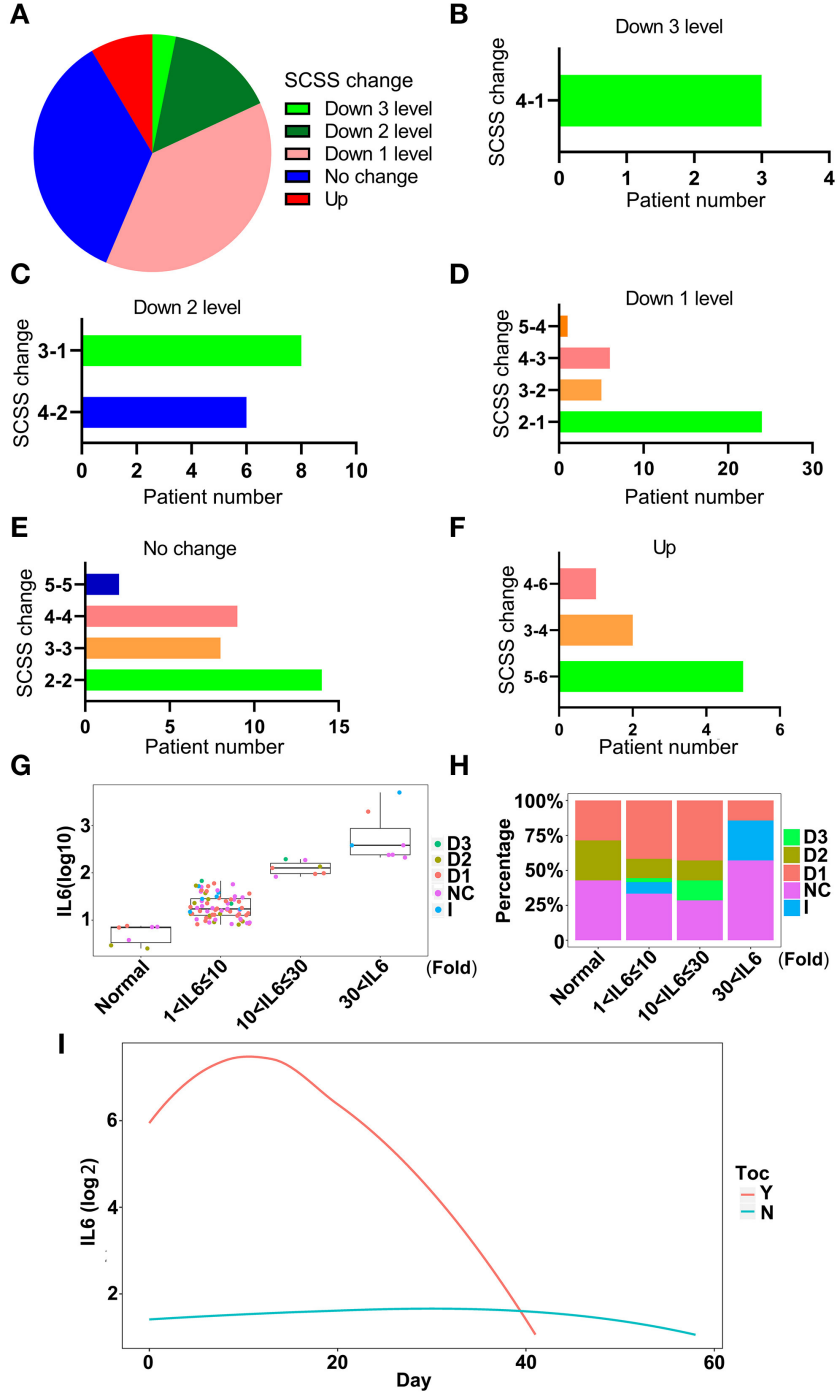
The effectiveness of tocilizumab relieves inflammation. **(A)** The changes in six-category scale score (SCSS) of patients treated with tocilizumab after 14 days. Down 3 levels, the SCSS decreased 3 levels; Down 2 levels, SCSS decreased 2 levels; Down 1 level, SCSS decreased 1 level; No change, SCSS not changed; Up, SCSS increased. **(B)** The number of SCSS changes that were decreased 3 levels. **(C)** The number of SCSS changes that were decreased 2 levels. **(D)** The number of SCSS changes that were decreased 1 level. **(E)** The number of SCSS with no change. **(F)** The number of SCSS changes that were increased 1 level. **(G)** The distribution of SCSS for each patient under different IL-6 levels. D1, SCSS decreased 1 level; D2, SCSS decreased 2 levels; D3, SCSS decreased 3 levels; I, SCSS increase; NC, SCSS not changed. **(H)** The percentage of patients with different SCSS and different IL-6 levels. D1, SCSS decreased 1 level; D2, SCSS decreased 2 levels; D3, SCSS decreased 3 levels; I, SCSS increased; NC, SCSS not changed. **(I)** The dynamic changes in IL-6 levels after treatment with tocilizumab. Y, IL-6 levels in patients treated with tocilizumab; N, IL-6 levels in patients without tocilizumab treatment.

To investigate whether tocilizumab could decrease the levels of IL-6 and relieve inflammation, the condition of patients after tocilizumab therapy for 14 days was assessed based on a six-category scale score (SCSS), and the therapeutic effect on each patient was evaluated. The effect of tocilizumab therapy varied in different patients (Figure 7A). A total of 56.3% of the patients had a decrease in the SCSS, which indicated improvement in the condition of the patients (Figure 7A). A total of 38.3% of patients were discharged within 14 days after treatment with tocilizumab (Figure 7A). To further illustrate the effect of tocilizumab, the changes in SCSS for each patient were analyzed. SCSS decreased from 2 to 1 in most of the patients in the improved patient group (Figures 7B–D). The largest number of patients, who were not improved after treatment with tocilizumab, had an SCSS of 2 (Figure 7E). Eight patients had an increase in the SCSS (Figures 7A,F). To further investigate the use of tocilizumab, the patients were divided into four groups according to IL-6 levels (Normal; 1 < IL-6 ≤ 10; 10 < IL-6 ≤ 30; and IL-6 > 30), and the prognoses of patients in each group were analyzed. Most patients treated with tocilizumab had an IL-6 level in the 10 < IL-6 ≤ 3 0 range and the SCSS of these patients were varied, which indicated that the prognoses of these patients were different (Figures 7G,H). Patients with an IL-6 level, 30-fold greater than the normal level, had a poor prognosis. There were no patients with a SCSS that decreased to two levels (Figures 7G,H). Analysis of dynamic changes in IL-6 levels after tocilizumab therapy indicated that the IL-6 levels of patients who received tocilizumab treatment were higher than in patients who were not treated with tocilizumab, which was consistent with the guidelines for the use of this treatment (Figure 7G). The level of IL-6 was increased due to tocilizumab treatment and then it decreased (Figure 7I). To further confirm the effect of tocilizumab treatment with IL-6, matched controls of patients treated with tocilizumab were selected (Supplementary Figures 5A,B), and comparison was done which also indicated the same trend of change in the IL-6 levels (Supplementary Figure 5C). But for the therapy endpoint of the tocilizumab treated patients, no improvement were found especially for the number of death case compared with group which were not treated by tocilizumab (Supplementary Figure 5D). These results indicated that therapy with tocilizumab could improve the inflammatory condition of patients, especially in patients with severe disease and high levels of IL-6.

### Convalescent Plasma Therapy Could Decrease IL-6 Levels

**Figure 8:**
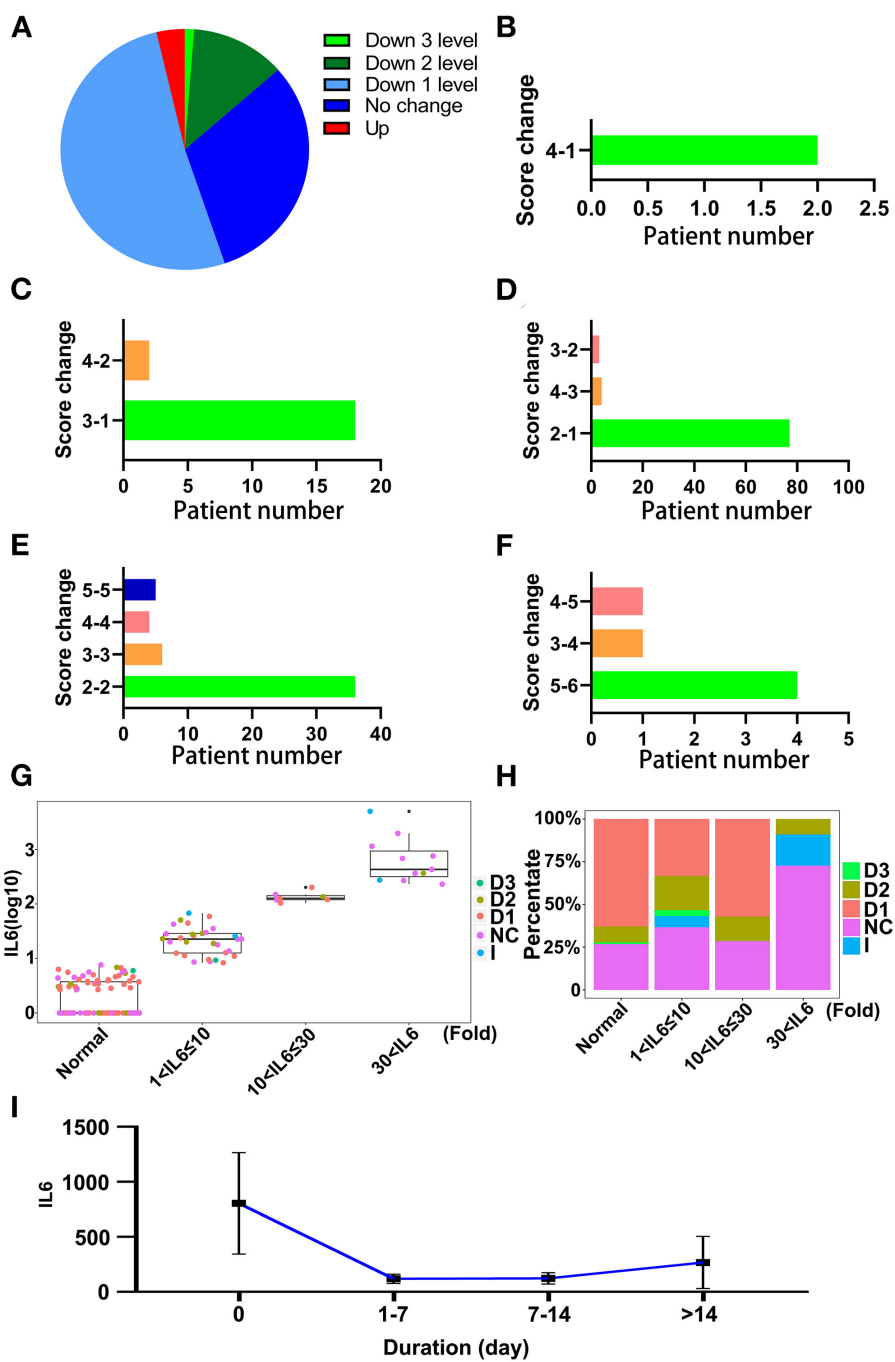
The effectiveness of convalescent plasma therapy to relieve inflammation. **(A)** The changes in SCSS of patients treated with convalescent plasma therapy within 14 days. Down 3 levels, SCSS decreased 3 levels; Down 2 levels, SCSS decreased 2 levels; Down 1 level, SCSS decreased 1 level; No change, SCSS not changed; Up, SCSS increased. **(B)** The number of SCSS changes decreased 3 levels. **(C)** The number of SCSS decreased 2 levels. **(D)** The number of SCSS changes decreased 1 level. **(E)** The number of SCSS with no change. **(F)** The number of SCSS changes increased 1 level. **(G)** The distribution of SCSS for each patient under different IL-6 levels. D1, SCSS decreased 1 level; D2, SCSS decreased 2 levels; D3, SCSS decreased 3 levels; I, SCSS increased; NC, SCSS not changed. **(H)** The percentage of patients with different SCSS with different IL-6 levels. D1, SCSS decreased 1 level; D2, SCSS decreased 2 levels; D3, SCSS decreased 3 levels; I, SCSS increased; NC, SCSS not changed. **(I)** The dynamic changes in IL-6 levels after treatment with convalescent plasma therapy (CPT).

Convalescent plasma therapy is an important medical treatment for patients with COVID-19. It also could relieve inflammation and alter the level of IL-6 (15). The effect of CPT was also evaluated using SCSS, and the results showed that the condition of over 65% of the patients treated with CPT had improved (Figure 8A). After treatment with CPT, 97 out of 163 patients treated with CPT were discharged within 14 days (Figures 8B–D). The largest percentage of patients who received CPT was the group that had a SCSS of 2 before therapy and were discharged later (Figure 8D). There were also a group of patients who were not response with the treatment of CPT and no SCSS change were found (Figure 8E). Although the condition of some patients became even worse, six patients out of the 163 patients who received CPT showed an increase in the SCSS (Figure 8F). This might be due to the fact that the patients had reached the end of the disease process at the time of the therapy. IL-6 levels in relation to CPT prognoses were also investigated. CPT treated patients were divided into four groups based on the IL-6 levels. Some patients showed a decrease of two points in SCSS, even in the group with the highest levels of IL-6 (Figures 8G,H). Considering the dynamic changes in IL-6 levels in the patients who underwent CPT therapy, it was found that CPT could decrease the degree of inflammation as indicated by a decrease in the IL-6 level (Figure 8I). In the subgroup of patients who received both tocilizumab and CPT treatment, about 16.6% of patients received CPT treatment (Supplementary Figure 6A). The change of IL-6 level of these patients also showed an uptrend after treatment with tocilizumab, but the duration of the increase was slightly shorter compared with patients who received tocilizumab treatment only (Supplementary Figure 6B). Based on these results, we deemed that patients with COVID-19, who received CPT therapy showed a decrease in the duration of the disease and a decrease in the degree of inflammation.

### Dynamic Changes in IL-6

**Figure 9:**
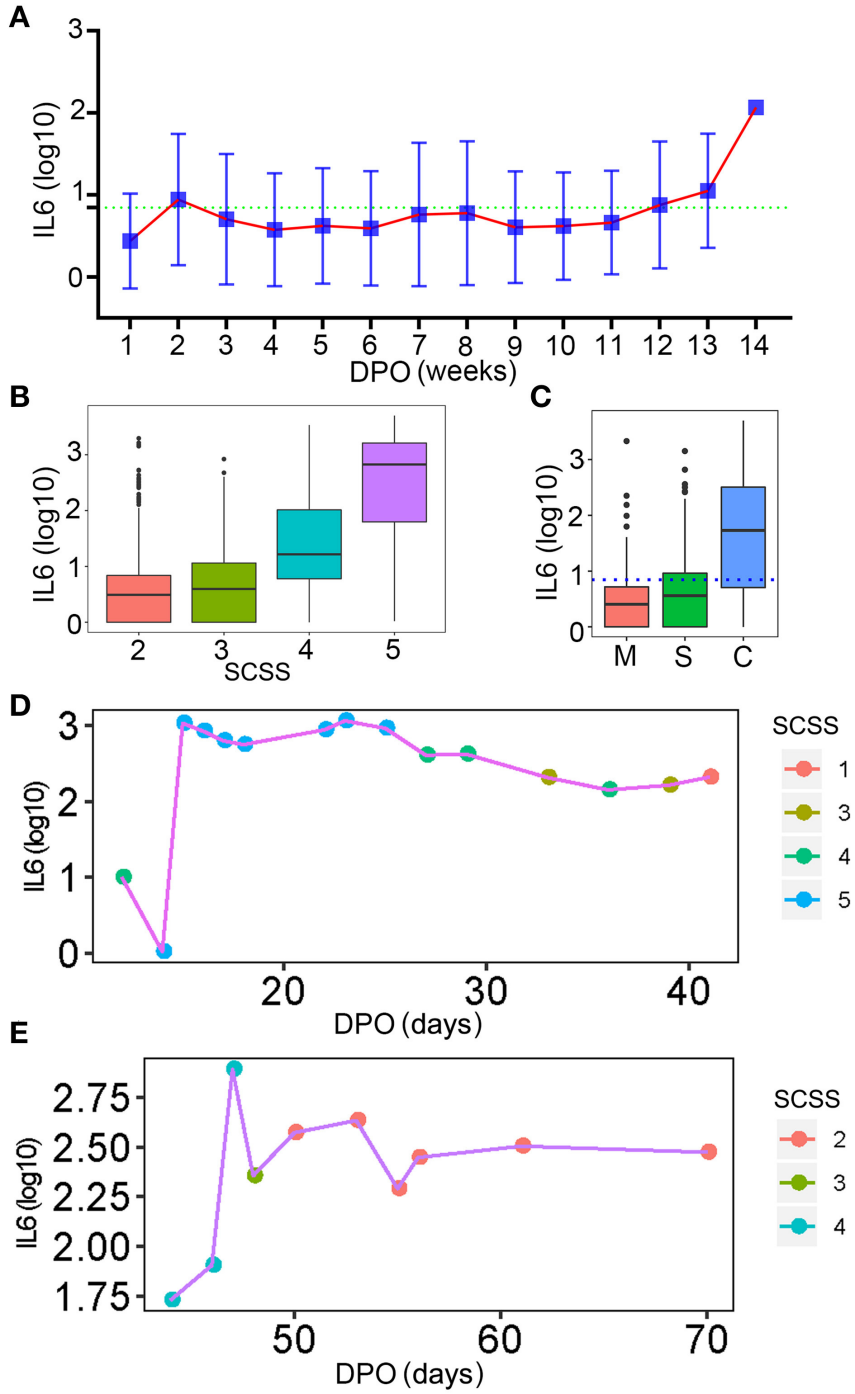
The dynamic changes in IL-6 levels. **(A)** The dynamic changes in IL-6 levels for all patients involved in this study. The normal level of IL-6 was indicated by the green dotted line. DPO, day post onset. **(B)** The IL-6 levels of patients with different levels of SCSS. **(C)** The IL-6 levels of patients who were discharged within 3 days. The normal level of IL-6 was indicated by the blue dotted line. M, mild patients; S, sever patients; C, critical patients. **(D,E)** The relationship between IL-6 levels and SCSS during the entire disease process for two patients. SCSS, six-category scale score.

As a key factor indicating the level of inflammation, dynamic changes in IL-6 levels during a SARS-CoV-2 infection process should be of high concern. The changes in IL-6 levels throughout the disease progression in patients with COVID-19 were analyzed. Fluctuation of IL-6 levels in the hospitalized patients was smooth at the 13th week after the onset of the disease (Figure 9A). This might be due to the long disease process and the inflammation condition becoming more critical. The level of IL-6 increased with the severity of infection in patients with COVID-19, which was indicated by SCSS (Figure 9B). The IL-6 levels of patients, who were discharged within 3 days, were also analyzed. As shown in Figure 9C, most patients who were classified as mild or severe had lower IL-6 levels compared with the critical group of patients. The IL-6 levels in critical patients with COVID-19 were much higher than the levels set as the cut-off value for IL-6 detection, which meant that this group of patients still had a high level of inflammation (Figure 9C). Although the changes in IL-6 levels of the hospitalized patients were not obvious, the dynamics of IL-6 in individual patients changed continuously (Figures 9D,E). Based on the analysis of changes in IL-6 levels during the infection stage, the inflammation condition of patients with COVID-19 was uncovered, especially the condition near the day of discharge.

## Discussion

The pandemic, COVID-19 has been overwhelming health systems worldwide. CSS, as the most important threat for critical patients, should be of concern. IL-6 plays a key role in inflammatory reactions. Investigation of the level of IL-6 during the entire SARS-Cov-2 infection process could provide a comprehensive understanding of the dynamic changes in inflammation, which could be beneficial for the monitoring and treating patients with COVID-19.

The health condition of patients with COVID-19 is an important influential factor in a SARS-Cov-2 infection (16–18). By analyzing the relationships among basic data obtained from patients with COVID-19, we found that age, sex, temperature, SpO_2_, and underlying diseases were all closely related to the inflammation level in patients with COVID-19 (Figures 1, 2). Male patients had higher levels of IL-6 compared to females. Patients who were elderly or had a higher temperature might suffer a more serious inflammatory reaction. As for underlying diseases, the inflammatory condition of patients with COVID-19 having a chronic obstructive pulmonary disease had a more severe infection compared to the other types of underlying diseases. This phenomenon might be due to the fact that COVID-19 attaches itself first to the lung. A SARS-CoV-2 infection could also cause several complications that could seriously affect the prognosis. Acute kidney injury (AKI), a critical complication of COVID-19, was assessed by many researchers (19–21). The condition of IL-6 with different levels of AKI was also analyzed in our study. Higher IL-6 levels were found in patients with severe AKI, which meant that the level of IL-6 could also indicate the progression of AKI (Supplementary Figure 3).

In addition to IL-6, several other cytokines were shown to play a role in COVID-19 disease. In our study, 12 types of cytokines were detected. Based on the correlation coefficients between IL-6 and the other cytokines, IL-10 was selected to be more closely related to IL-6 (Figure 4). Combining these two cytokines, the predictive capability was increased slightly. These results further demonstrated the reliability of IL-6 as an indicator of inflammation and the progression of the disease. The key role of IL-6 in COVID-19 was also emphasized, and it could be used as a therapy target in the treatment of patients with COVID-19.

Tocilizumab, as a treatment targeting IL-6 receptor, has been reported to improve the clinical outcomes of patients with severe COVID-19 (22). Consistent with the previous report, our findings also indicated the effectiveness of tocilizumab in COVID-19 treatment (22–24). Patients who received tocilizumab therapy showed diverse changes in SCSS (Figure 7). Patients with different IL-6 levels before treatment with tocilizumab showed improvement in the prognosis with treatment. Based on our results, patients with IL-6 levels 30-fold above the normal condition had a poor prognosis. This result indicated that the use of tocilizumab as a therapy for COVID-19 is acceptable. Importantly, we found that the level of IL-6 in patients who received tocilizumab treatment was increased at the beginning of the therapy procedure (Figure 7). The transient increase of IL-6 after the treatment of tocilizumab, were also reported by other researchers (25). This phenomena might be caused by the free IL-6 in plasma, as its receptors have been blocked by tocilizumab (26, 27), and this has also decreased the efficiency of IL-6 degeneration. The increase of IL-6 might be the consequence of disorder in patients who already had high levels of IL-6 (28). In order to compete with tocilizumab that binds with the IL-6 receptor, the body releases more IL-6. There were also reports that showed that the tocilizumab therapy might worsen the symptoms, especially by increasing the risk of viral and bacterial infection (28). This increased risk caused by the weakening of innate immunity in which IL-6 performed critical roles. This finding could also provide a guide for monitoring the patients treated with tocilizumab. The outcomes of patients treated with tocilizumab, as indicated by SCSS, reflected the effectiveness; over 50% of patients showed improved clinical symptoms in 14 days. There were also retrospective studies that reported that the treatment of tocilizumab might be associated with low risk of death or intubation in patients with severe COVID-19 infection (22, 27, 29–31). However, the observational nature of these studies might hamper the assessment of the effect of treatment with tocilizumab (32). For the final treatment, outcomes of patients treated with tocilizumab were analyzed; and cases of death were even more than the control group. This phenomenon was also reported by a randomized, double-blind, placebo-controlled trial, which demonstrated the non-effectiveness of tocilizumab in preventing death in moderately ill and hospitalized patients with COVID-19 (33). In our study, we deemed that this might be caused by the condition of the patients who received the tocilizumab treatment. This group of patients had higher levels of IL-6, indicating that the high level of inflammation was caused by infection.

Compared to treatment with tocilizumab, CPT, another specific treatment for COVID-19 was also highly effective. The decrease of SCSS in patients treated with CPT was better than in patients treated with tocilizumab; the SCSS was found to be reduced even in patients with high levels of IL-6. As indicated by the decrease of SCSS, the clinical symptoms of patients treated with CPT were improved, consistent with many studies (34–36). Multiple clinical laboratory test items were also improved after the transfusion of convalescent plasma, such as increase of SpO_2_, lymphocyte counts, improvement of CRP, and liver function (34). Consistent with the decrease of IL-6 level in our study, these results suggest that inflammation in patients with COVID-19 were alleviated through the neutralizing SARS-Cov-2 antibodies contained in convalescent plasma. More importantly, we also found that the IL-6 level of patients who received both CPT and tocilizumab treatment could decrease in a shorter period of time compared with patients who were treated only with tocilizumab. We inferred that the CPT could control the inflammation quickly and less IL-6 would be released into the plasma.

Based on the discharge criteria, patients with two consecutive negative results on nuclear acid detection tests were discharged. Our study demonstrated the inflammatory condition of patients who could be discharged. Patients who matched the standard of discharge also had different inflammatory conditions, especially patients with a history of critical COVID-19 infection. They still had a high level of IL-6 and the inflammatory reaction of these patients was monitored continually. This means that, although the SARS-CoV-2 virus may be cleared in patients with COVID-19, anti-inflammation therapy should be performed further to help with recovery, especially for critical patients.

There were some limitations in this study. The design of this study was correlational, and the mechanisms under the relationship between IL-6 and several aspects of clinical features of the patients should be investigated further. All patients came from one specialist hospital; and the diversity of patients was limited. Our study is also limited by its non-randomized retrospective design. Besides, it was a single observation study, and a significant bias could have possibly existed.

## Conclusions

In conclusion, our study provided a comprehensive view of the cytokine IL-6. It demonstrated a close relationship between IL-6 levels and health conditions of patients with COVID-19. Tocilizumab and CPT treatments, the two important therapy methods for COVID-19, decreased the level of IL-6 and relieved inflammation. More importantly, we found that the treated patients with IL-6 levels 30-fold higher than normal had a poor prognosis. Discharged patients who had COVID-19 might still have a high level of IL-6, and more attention should be paid to the inflammatory levels of patients, even after the discharge.

## Data Availability Statement

The original contributions presented in the study are included in the article/Supplementary Material, further inquiries can be directed to the corresponding author.

## Ethics Statement

This study was approved by the Medical Ethical Committee of Wuhan Huoshenshan Hospital of China, and written informed consent was obtained from each patient.

## Author Contributions

JW, JS, YH, and QQ contributed to the data analysis and wrote the manuscript. WD, BH, RP, and JZhao contributed to data analysis and figure generation. TL, YG, YY, QW, WJ, and JZhan contributed to the data collection. MZ, NL, and WL provided advice during manuscript writing. XX conceived the study. All authors contributed to the article and approved the submitted version.

## Conflict of Interest

The authors declare that the research was conducted in the absence of any commercial or financial relationships that could be construed as a potential conflict of interest.

## Supplementary Material

Supplementary Figure 1 The distribution of ages and IL-6 levels for all patients. The age distribution of the patients.

Supplementary Figure 2 The ROC curve for 12 cytokines. The predictive ability of 12 cytokines is shown by an ROC curve. The tested cytokines and chemokines were listed with corresponding color lines.

Supplementary Figure 3 The relationship between IL-6 levels and indices related to different injured organs. **(A)** The relationship between IL-6 levels and biochemical indices related to myocardial injury. CK, creatine kinase; LDH, lactate dehydrogenase; HBDH, α-hydroxybutyrate dehydrogenase; CK-MB, creatine kinase isoenzymes; >30 fold, the biochemical index 30-fold higher than normal level; 1–30 fold, the biochemical index located in 1–30 fold of normal level; Normal, the biochemical index in normal level. **(B)** The relationship between IL-6 levels and routine blood test indices related to myocardial injury. CRP, C-reactive protein; UCRP, hypersensitive C-reactive protein; >30-fold, the biochemical index 30-fold higher than normal level; 1–30 fold, the biochemical index located in 1–30 fold in the normal level; Normal, the biochemical index in normal level. **(C)** The relationship between IL-6 levels and BNP. BNP, B-type natriuretic peptide; >30-fold, the biochemical index 30-fold higher than the normal level; 1–30 fold, the biochemical index located in 1–30 fold of normal level; Normal, the biochemical index in normal level. **(D)** The relationship between IL-6 and biochemical indices related to kidney injury. Urea, urea nitrogen; Cre, creatinine; UA, uric acid; CysC, cystatin C; CO_2_, carbon dioxide; >30-fold, the biochemical index 30-fold higher than the normal level; 1–30 fold, the biochemical index located in 1–30 fold in the normal level; Normal, the biochemical index in the normal level. **(E)** The relationship between IL-6 levels and routine blood test indices related to kidney injury. LYM%, lymphocyte percentage; MONO%, monocytes percentage; EOS%, eosinophil percentage; >0-fold, the biochemical index 30-fold higher than the normal level; 1–30 fold, the biochemical index located in 1–30 fold in the normal level; Normal, the biochemical index in the normal level. **(F)** The IL-6 distribution for different acute kidney injury levels. AKI: acute kidney injury. **(G)** The relationship between IL-6 and routine blood test indices related to liver injury. RBC, red blood cell; HCT, red blood cell specific volume; MCV, mean corpuscular volume; MCHC, mean corpuscular hemoglobin concentration; RDW-CV, red blood cell distribution width; PLT, blood platelet; MPV, mean platelet volume; BASO%, basocyte percentage; NEUT#, Neutrophil count; >30-fold, the biochemical index 30-fold higher than the normal level; 1–30 fold, the biochemical index located in 1–30 fold in the normal level; Normal, the biochemical index in the normal level. **(H)** The relationship between IL-6 levels and biochemical indices related to liver injury. ALT, glutamic-pyruvic transaminase; AST, glutamic oxaloacetic transaminase; TP, total protein; ALB, albumin; GLB, globulin; A/G, albumin/globulin ration; TBIL, total bilirubin; DBIL, bilirubin direct; IBIL, indirect bilirubin; TBA, total bile acid; ALP, alkaline phosphatase; GGT, gamma glutamyl transpeptidase; >30-fold, the biochemical index 30-fold higher than the normal level; 1–30 fold, the biochemical index located in 1–30 fold in the normal level; Normal, the biochemical index in the normal level.

Supplementary Figure 4 The relationship between IL-6 levels and coagulation indices. Changes in coagulation indices shown for different levels of IL-6. FIB, fibrinogen; APTT, activated partial thromboplastin time; PT, plasma prothrombin time; plasma thrombin time; INR, international normalized ratio; PT%, prothrombin activity; Normal, IL-6 level at the normal level; 1 < IL-6 <30-fold, IL-6 level located in 1–30 fold of normal IL-6 level; IL-6 > 30-fold, IL-6 level higher than 30-fold in the normal level.

Supplementary Figure 5 The effect of tocilizumab treatment. **(A)** The distribution of patients in two age groups. Toc+, patients with tocilizumab treatment; Toc-, patients without tocilizumab treatment. **(B)** The distribution of patients according to their gender. Toc+, patients with tocilizumab treatment; Toc-, patients without tocilizumab treatment. Female, female patients; Male, male patients. **(C)** The trend of IL-6 after tocilizumab treatment. Toc+, patients with tocilizumab treatment; Toc-, patients without tocilizumab treatment. **(D)** The comparison of final treatment outcomes of tocilizumab treated and not treated groups which were indicated by SCSS. Toc+, patients with tocilizumab treatment; Toc-, patients without tocilizumab treatment; 1–6, the level of SCSS.

Supplementary Figure 6 The IL-6 level of patients treated with CPT and tocilizumab. **(A)** The distribution of patients who received two kinds of treatment. **(B)** Comparison of IL-6 trend of patients between treatment strategies. CPT + TOC, patients treated with both convalescent plasma therapy (CPT) and tocilizumab; TOC, patients treated by tocilizumab only.
