# Supplementary figures and images for: Upregulated IL-6 Indicates a Poor COVID-19 Prognosis: A Call for Tocilizumab and Convalescent Plasma Treatment
Source: Front Immunol. 2021 Mar 4;12:598799. doi: 10.3389/fimmu.2021.598799 (PMC7969719; doi:10.3389/fimmu.2021.598799)

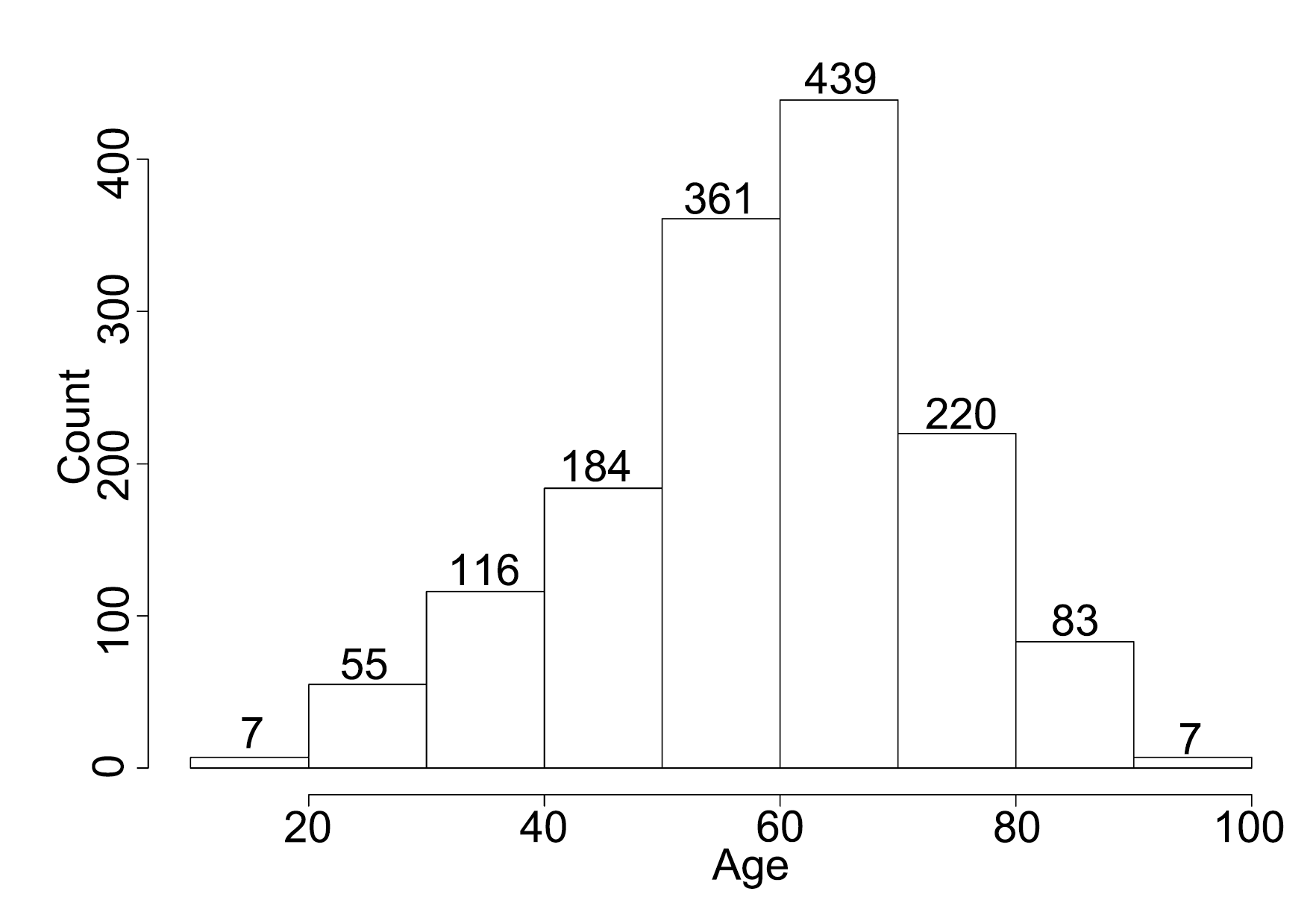

Supplement: Supplementary Figure 1 — The distribution of ages and IL-6 levels for all patients. The age distribution of the patients. [file Image_1.TIF]

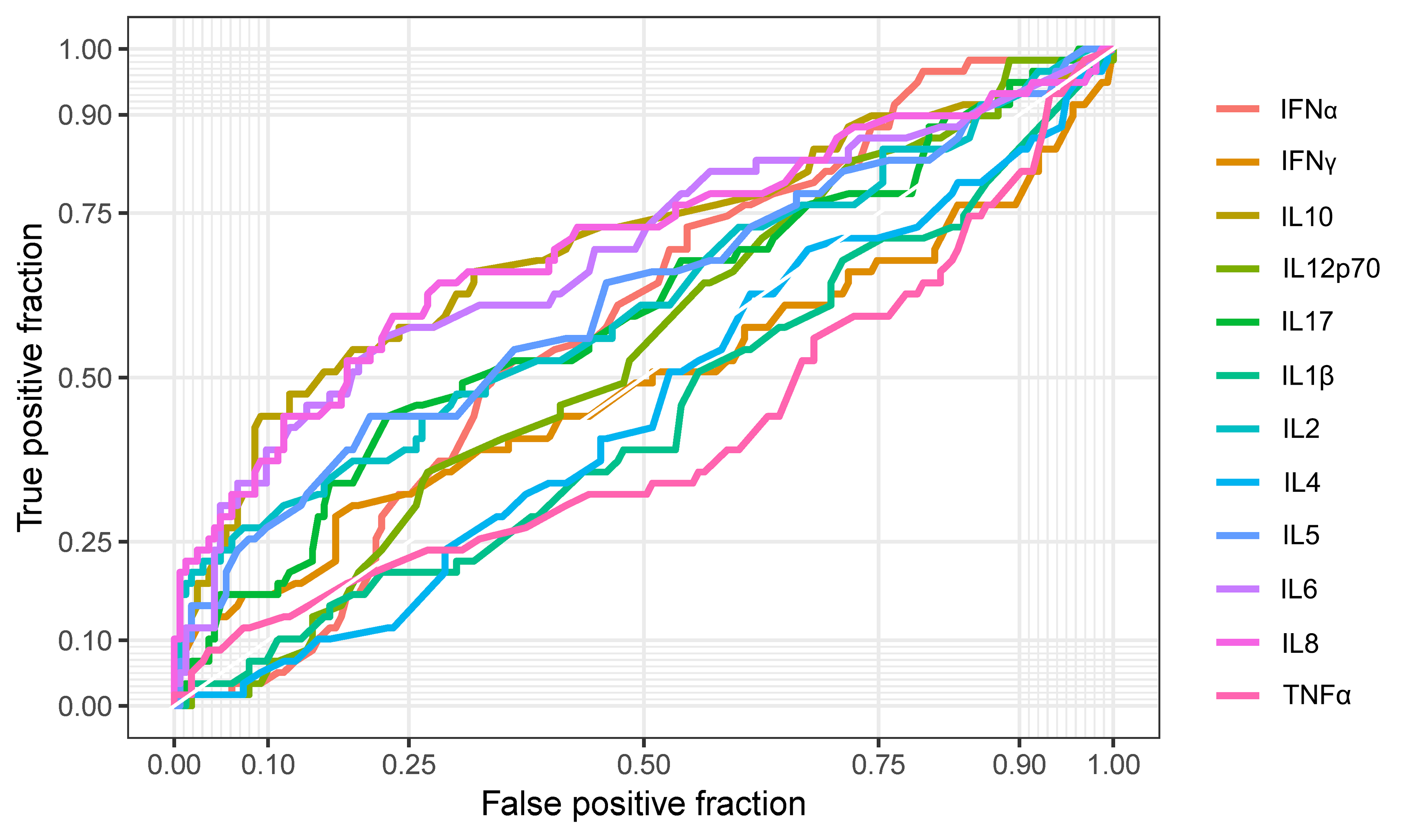

Supplement: Supplementary Figure 2 — The ROC curve for 12 cytokines. The predictive ability of 12 cytokines is shown by an ROC curve. The tested cytokines and chemokines were listed with corresponding color lines. [file Image_2.TIF]

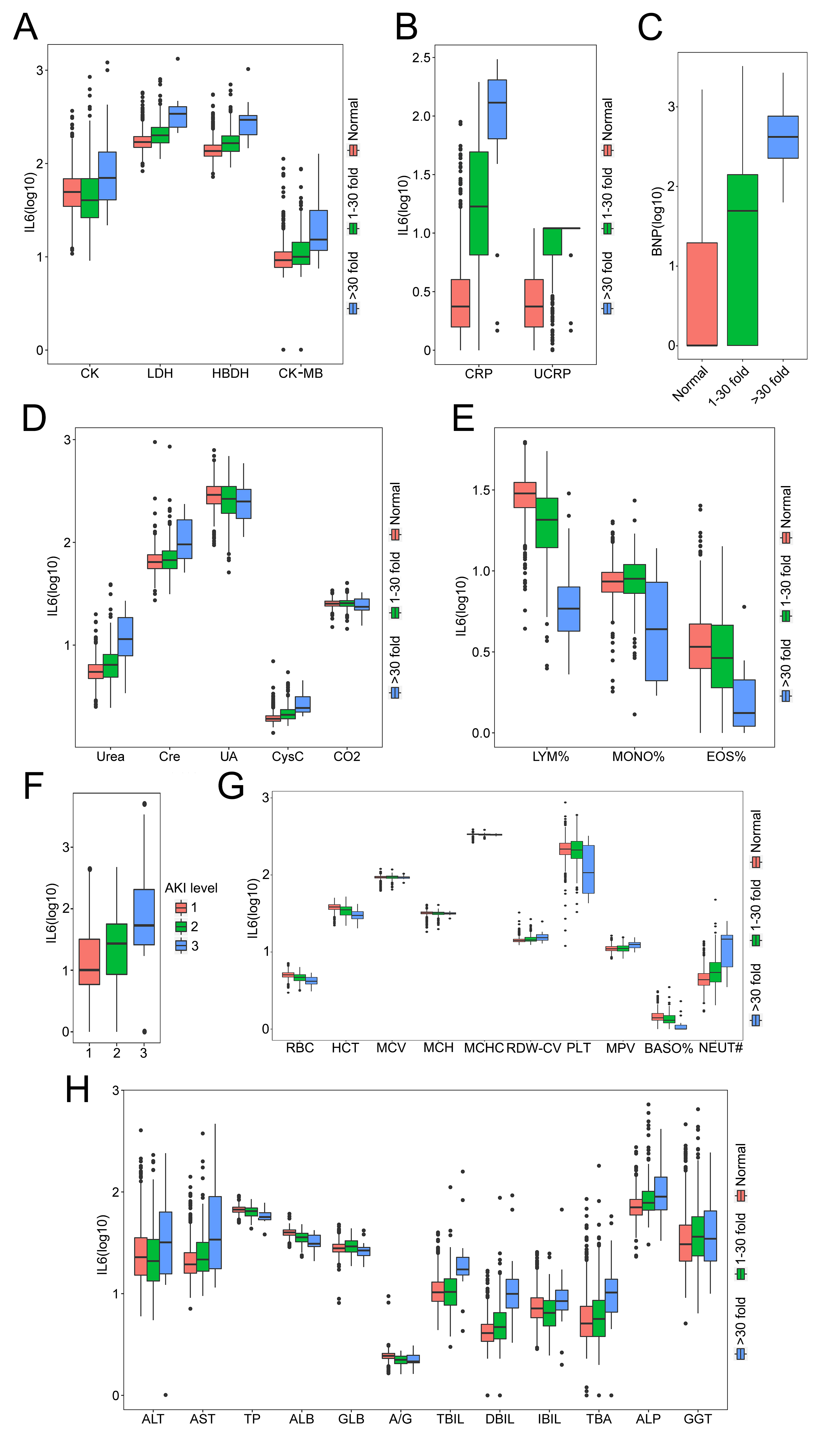

Supplement: Supplementary Figure 3 — The relationship between IL-6 levels and indices related to different injured organs. (A) The relationship between IL-6 levels and biochemical indices related to myocardial injury. CK, creatine kinase; LDH, lactate dehydrogenase; HBDH, α-hydroxybutyrate dehydrogenase; CK-MB, creatine kinase isoenzymes; >30 fold, the biochemical index 30-fold higher than normal level; 1–30 fold, the biochemical index located in 1–30 fold of normal level; Normal, the biochemical index in normal level. (B) The relationship between IL-6 levels and routine blood test indices related to myocardial injury. CRP, C-reactive protein; UCRP, hypersensitive C-reactive protein; >30-fold, the biochemical index 30-fold higher than normal level; 1–30 fold, the biochemical index located in 1–30 fold in the normal level; Normal, the biochemical index in normal level. (C) The relationship between IL-6 levels and BNP. BNP, B-type natriuretic peptide; >30-fold, the biochemical index 30-fold higher than the normal level; 1–30 fold, the biochemical index located in 1–30 fold of normal level; Normal, the biochemical index in normal level. (D) The relationship between IL-6 and biochemical indices related to kidney injury. Urea, urea nitrogen; Cre, creatinine; UA, uric acid; CysC, cystatin C; CO2, carbon dioxide; >30-fold, the biochemical index 30-fold higher than the normal level; 1–30 fold, the biochemical index located in 1–30 fold in the normal level; Normal, the biochemical index in the normal level. (E) The relationship between IL-6 levels and routine blood test indices related to kidney injury. LYM%, lymphocyte percentage; MONO%, monocytes percentage; EOS%, eosinophil percentage; >0-fold, the biochemical index 30-fold higher than the normal level; 1–30 fold, the biochemical index located in 1–30 fold in the normal level; Normal, the biochemical index in the normal level. (F) The IL-6 distribution for different acute kidney injury levels. AKI: acute kidney injury. (G) The relationship between IL-6 and r [file Image_3.TIF]

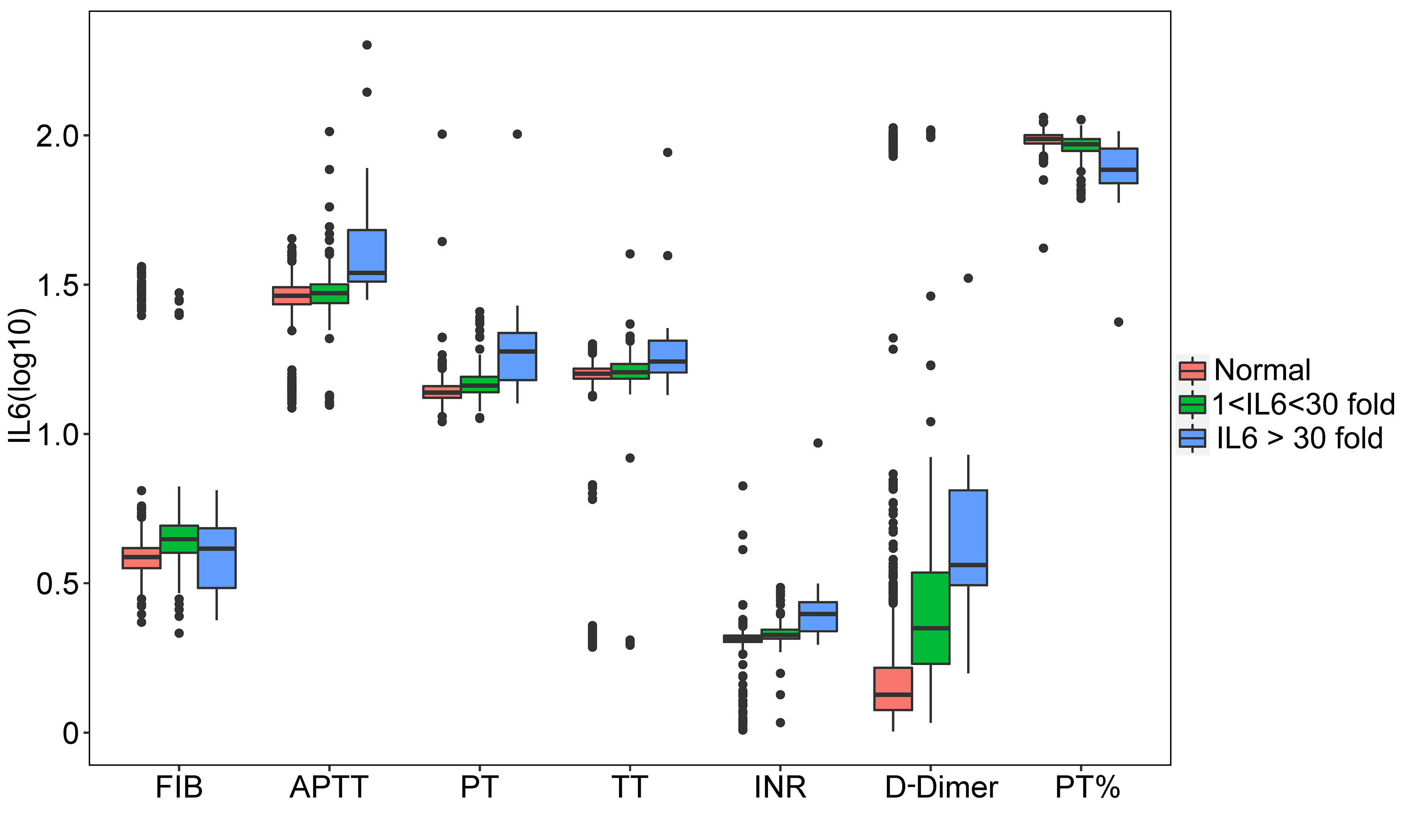

Supplement: Supplementary Figure 4 — The relationship between IL-6 levels and coagulation indices. Changes in coagulation indices shown for different levels of IL-6. FIB, fibrinogen; APTT, activated partial thromboplastin time; PT, plasma prothrombin time; plasma thrombin time; INR, international normalized ratio; PT%, prothrombin activity; Normal, IL-6 level at the normal level; 1 < IL-6 <30-fold, IL-6 level located in 1–30 fold of normal IL-6 level; IL-6 > 30-fold, IL-6 level higher than 30-fold in the normal level. [file Image_4.TIFF]

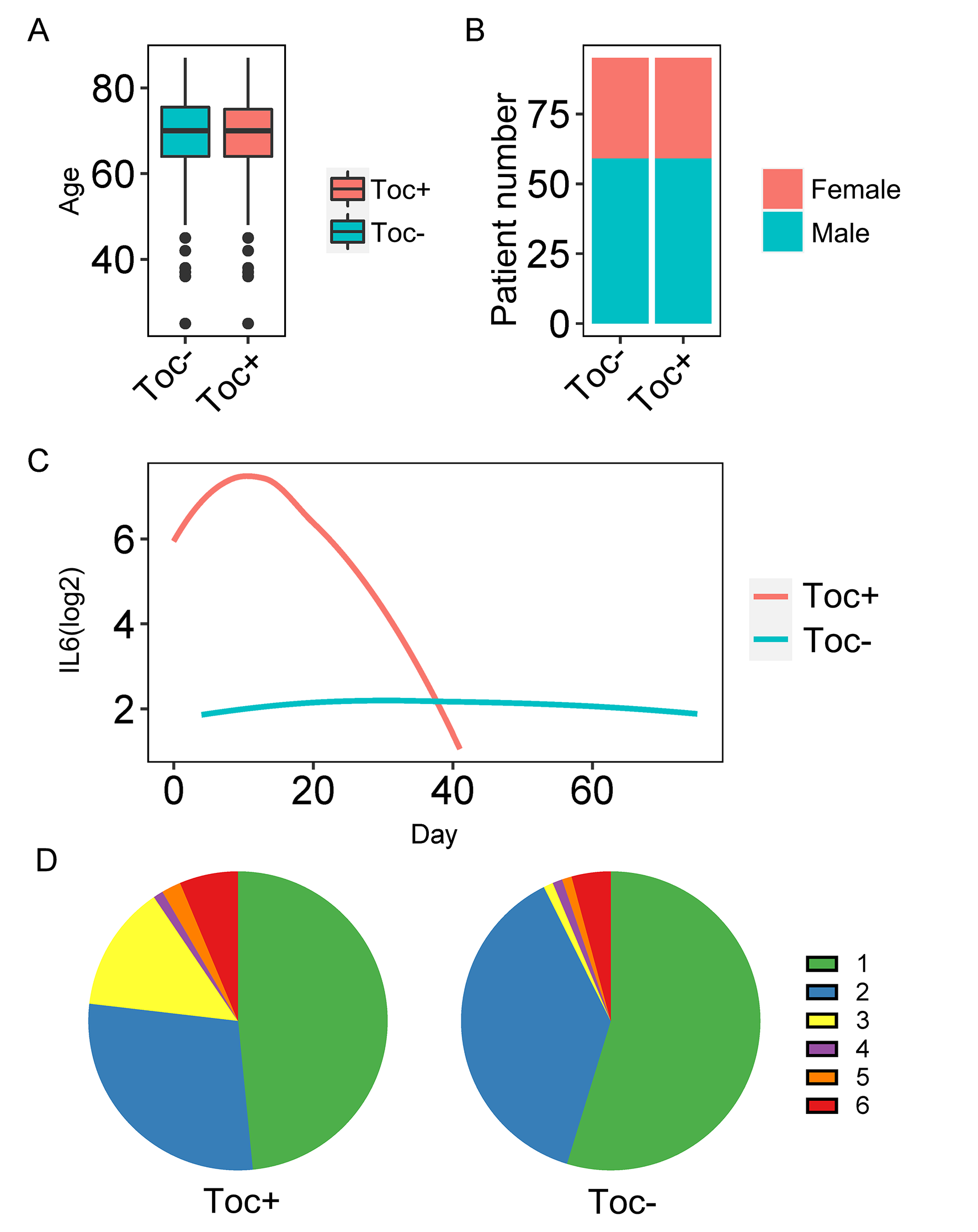

Supplement: Supplementary Figure 5 — The effect of tocilizumab treatment. (A) The distribution of patients in two age groups. Toc+, patients with tocilizumab treatment; Toc-, patients without tocilizumab treatment. (B) The distribution of patients according to their gender. Toc+, patients with tocilizumab treatment; Toc-, patients without tocilizumab treatment. Female, female patients; Male, male patients. (C) The trend of IL-6 after tocilizumab treatment. Toc+, patients with tocilizumab treatment; Toc-, patients without tocilizumab treatment. (D) The comparison of final treatment outcomes of tocilizumab treated and not treated groups which were indicated by SCSS. Toc+, patients with tocilizumab treatment; Toc-, patients without tocilizumab treatment; 1–6, the level of SCSS. [file Image_5.TIF]

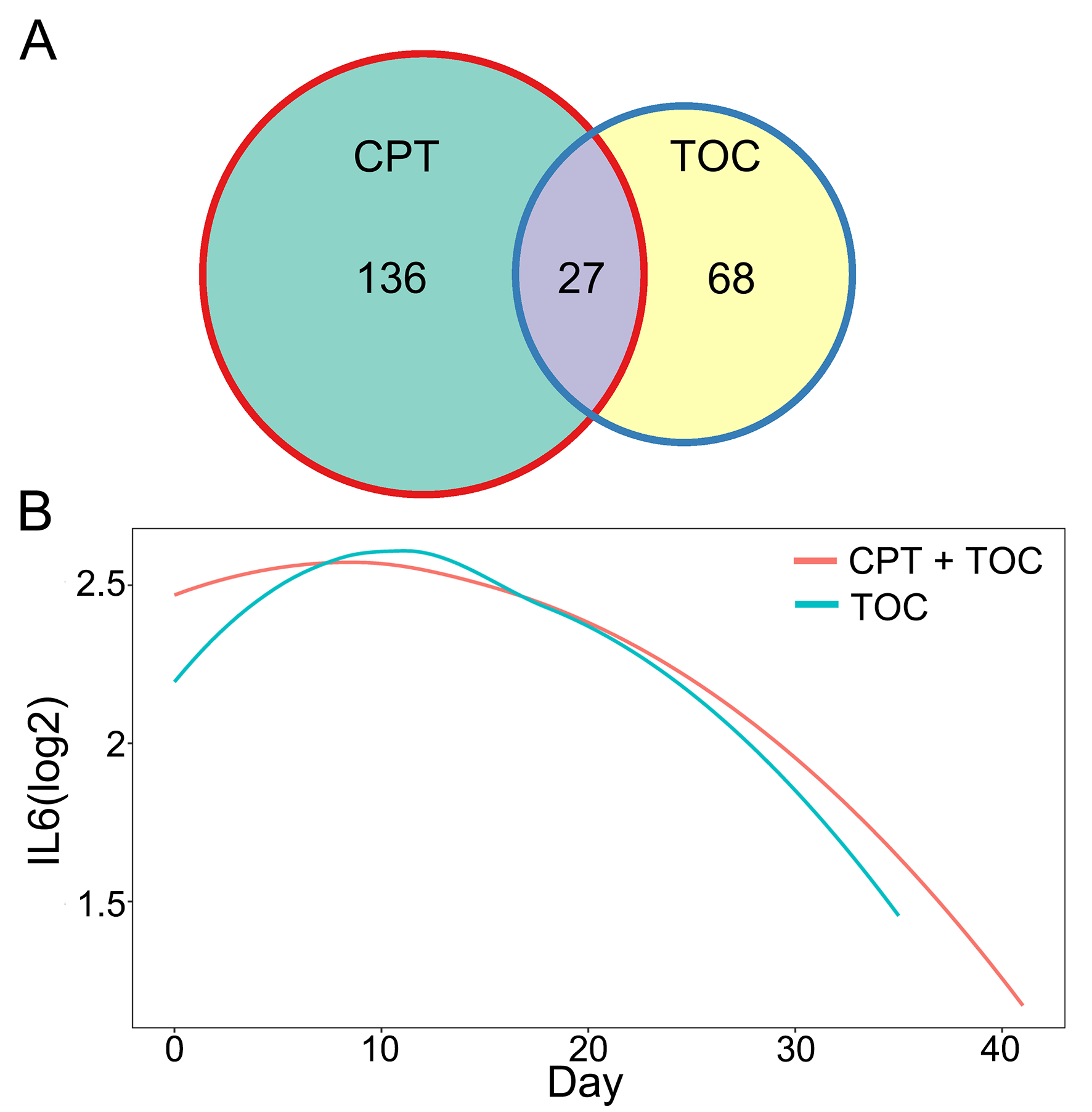

Supplement: Supplementary Figure 6 — The IL-6 level of patients treated with CPT and tocilizumab. (A) The distribution of patients who received two kinds of treatment. (B) Comparison of IL-6 trend of patients between treatment strategies. CPT + TOC, patients treated with both convalescent plasma therapy (CPT) and tocilizumab; TOC, patients treated by tocilizumab only. [file Image_6.TIF]
